# 

**Published:** 1948

**Authors:** 


### CONTENTS.

| | Page |
|---|---|
| Edward Jenner. By T. Milling, M.B., Ch.B. | 1 |
| The Diagnostic Work of a Surgical Thoracic Unit, with special reference to Intra-thoracic Suppuration and Bronchogenic Carcinoma. By R. H. R. Belsey, M.B., M.S., F.R.C.S. | 10 |
| A Case of Macrocheilia. By D. C. Bodenham, M.B., F.R.C.S. | 15 |
| Studies of the Renal Circulation. (A Review). | 16 |
| Alfred, the Zoo Gorilla. By Dr. R. C. Clarke | 19 |
| Reviews of Books | 21 |
| Editorial Notes | 23 |
| Editor's Report | 24 |
| Obituaries: Thomas Carwardine, M.S., F.R.C.S. | 27 |
| C. A. Moore, M.S., F.R.C.S. | 27 |
| Meetings of Societies | 28 |
| The Medical Library of the University of Bristol | 29 |
| Publications Received | 31 |
| Local Medical Notes | 32 |

